# Atypical “accelerated” chronic lymphocytic leukemia with abnormal lymphocyte chromatin clumping, bone involvement, and exceptional response to Imbruvica

**DOI:** 10.1002/cnr2.1601

**Published:** 2022-01-24

**Authors:** Leonid L. Yavorkovsky

**Affiliations:** ^1^ Oncology Division Kaiser Permanente San Jose Medical Center San Jose California USA

**Keywords:** accelerated, chromatin, leukemia, lymphocytes, pelgeroid

## Abstract

**Background:**

The “accelerated” chronic lymphocytic leukemia (aCLL) is a relatively rare form of CLL progression. The expanded proliferation centers in aCLL have been associated with adverse prognostic features and propensity to more aggressive behavior with shorter survival.

**Case:**

An atypical case of aCLL with distinct features is described. A 66‐year‐old female presented with a marrow replacing process associated with multiple osseous metastases and trivial lymphadenopathy. Bone biopsy revealed an unspecified low‐grade B cell lymphoproliferative disorder that demonstrated a suboptimal response to standard chemotherapy. Subsequent lymph node biopsy demonstrated findings consisted with aCLL. The distinguishing features of the case were, in addition to bone involvement, the lagging peripheral lymphocytosis and a striking pattern of the chromatin clumping with a prominent “shattered” appearance reminiscent of Pelger‐Huet‐like dysplastic anomaly. A targeted next‐generation sequencing (NGS) assay detected pathogenic mutations in *TP53* and *SF3B1*. In contrast to chemotherapy, the case demonstrated an excellent response to imbruvica.

**Conclusion:**

The noted peculiarities could potentially distinguish this case as a novel, rare variant of aCLL.

## 1 INTRODUCTION

“Accelerated” CLL (aCLL) is a relatively rare form of CLL progression. Although distinguished as early as 1988,^1^ only in recent years has it acquired more distinct histological, clinical and prognostic attributes.^2^ At present, the diagnosis of aCLL is defined by the presence of expanded and/or highly active proliferation centers (broader than a 20× field) and high proliferation rate (either >2.4 mitoses/proliferation center or Ki‐67 > 40%/proliferation center). The aCLL patients tend to display higher serum lactate dehydrogenase levels, more frequently elevated ZAP‐70 and a more aggressive course compared to a common CLL.^1^, ^2^

The incidence of aCLL could be underestimated because the lymphoid tissue biopsy that is sine qua non of the diagnosis is not commonly performed even in the presence of growing lymph nodes. As the result, clinical and phenotypic aspects of aCLL remain understudied. This case, despite histologic features compatible with aCLL, exhibited no peripheral lymphocytosis, a few immunophenotypic irregularities and hitherto unreported bone involvement. After developing a leukemic phase, malignant lymphocytes exhibited a striking morphologic appearance, which along with an excellent Imbruvica effect are expanding our knowledge of this still poorly recognized form of CLL progression.

### 1.1 Case report

**FIGURE 1:**
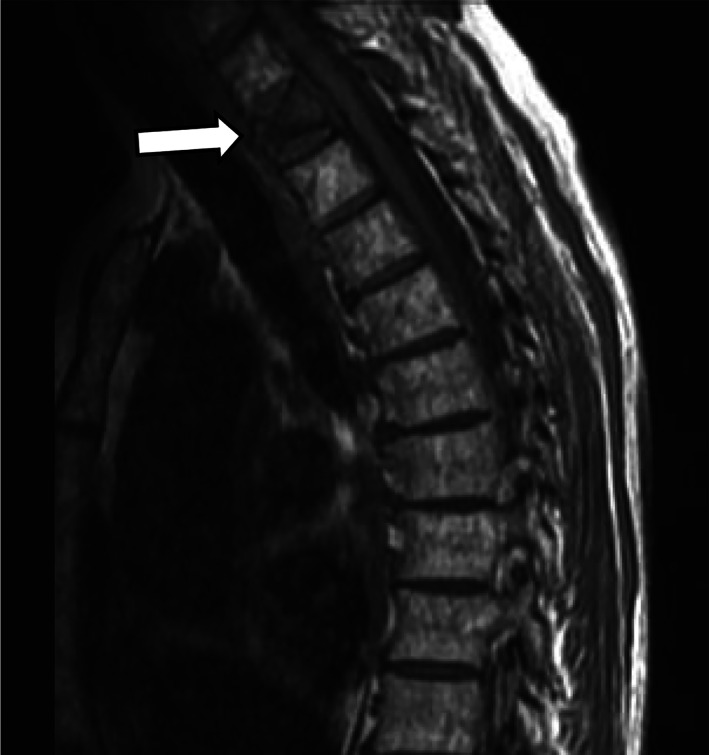
Magnetic resonance imaging of the spine showing pathologic fracture at T2 (arrow)

**FIGURE 2:**
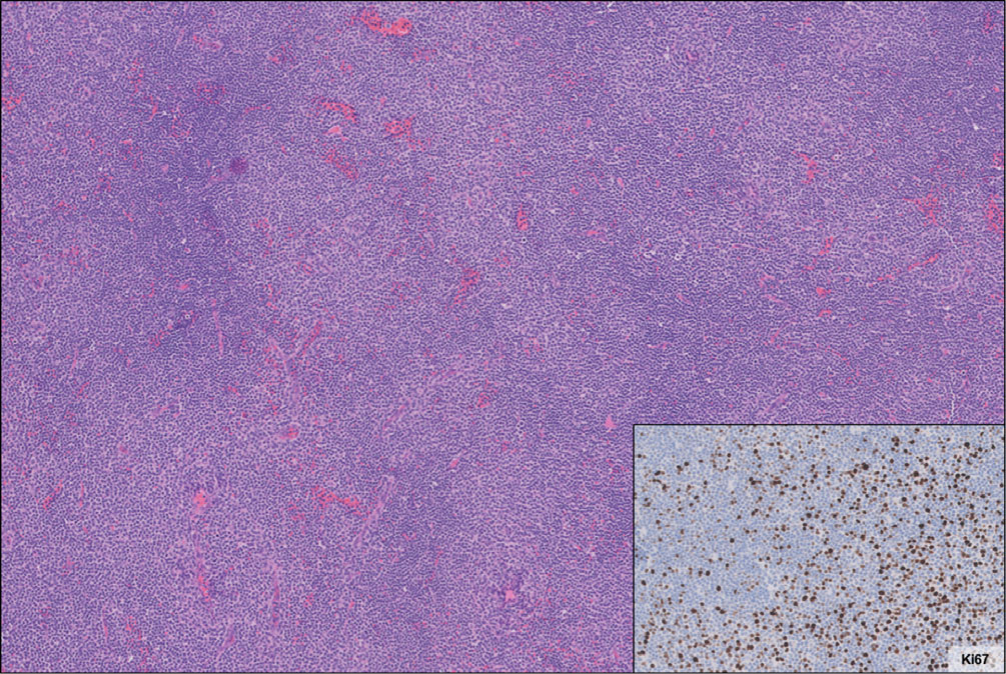
Histologic characteristics of excised axillary lymph node. Histologic sections show effacement of normal architecture by a proliferation of lymphocytes with coarse chromatin with multiple areas of pallor consistent with proliferation centers that become confluent in areas. The Ki67 proliferation index is approximately 5%–10% in the areas of smaller lymphocytes, with increased proliferation index of approximately 40% in the expanded proliferation centers (inset, bottom right)

A 66‐year‐old female was incidentally found to have a marrow replacing process involving clivus and C1 vertebral body on magnetic resonance imaging. Additionally, osseous metastases and pathologic fracture at T2 were noted (shown in Figure 1). A positron emission tomography (PET) showed scattered fluorodeoxyglucose (FDG)‐avid lesions in the T2, right 6th rib, left humerus, sacrum, pelvis, diffuse small lymphadenopathy and normal spleen. On biopsy (01/2020), the T2 lesion demonstrated a low‐grade B cell lymphoproliferative disorder characterized by diffuse proliferation of monotonous small to slightly large lymphoid cell with small nuclei, irregular nuclear contours, condensed chromatin, inconspicuous nucleoli, and scant cytoplasm. The cells were expressing PAX5, CD23 and CD79a, while negative for CD5, CD10, CD20, and CD22. Rearrangements of MYC (8q24), BCL6 (3q27) and IGH‐BCL2 fusion, t(14;18), were negative by fluorescent in‐situ hybridization analysis. Blood counts were unremarkable with normal differential. LDH was 233 U/L (reference range < =270 U/L). Hepatitis B, C, HIV and serum protein electrophoresis were normal. Because of extensive bone involvement by a B cell lymphoma‐type disorder, the patient received four cycles of cyclophosphamide, doxorubicin, vincristine and prednisone (CHOP) chemotherapy with improvement in some bones but worsening in others and increasing activity in the right axillary lymph nodes on a PET scan. The right axillary lymph node was biopsied (08/2020) and revealed histologic findings most consistent with “accelerated” chronic lymphocytic leukemia/small lymphocytic lymphoma due to an increase in confluent proliferation centers (shown in Figure 2) and Ki67 proliferation greater than 40% of the atypical lymphocytes within a proliferation center (shown in Figure 2, inset). The lymphocytes were positive for PAX5, Bcl2, Bcl6, MUM1, LEF1, CD19, CD23, CD38 (dim), CD79a and CD200, while negative for CD5, CD10, CD20, CD22, CD30, CD34, SOX11, and MYC. EBV was negative by in‐situ hybridization stain. Flow cytometry showed a lambda‐restricted B‐cell population that was equivocal for CD5. LDH was 343 U/L. Targeted next‐generation sequencing (NGS) assay was performed using a clinically validated targeted NGS panel (Heme Stanford Actionable Mutation Panel for Hematopoietic and Lymphoid Malignancies [Heme‐STAMP]). The Heme‐STAMP assay detected pathogenic mutations in *TP53* p.Cys141Tyr and *SF3B1* p.His662Leu shown in Table 1.

**TABLE 1:** Somatic mutations identified by next‐generation sequencing

Gene	Position	Variant allele frequency, %	Nucleotide change	Type of mutation	Amino acid change	Pathway
*TP53*	chr17:g.7578508	84	C > T	Missense	C141Y	Tumor suppressor; induces growth arrest or apoptosis
*SF3B1*	chr2:g.198267372	48	T > A	Missense	H662L	mRNA splicing and processing

**FIGURE 3:**
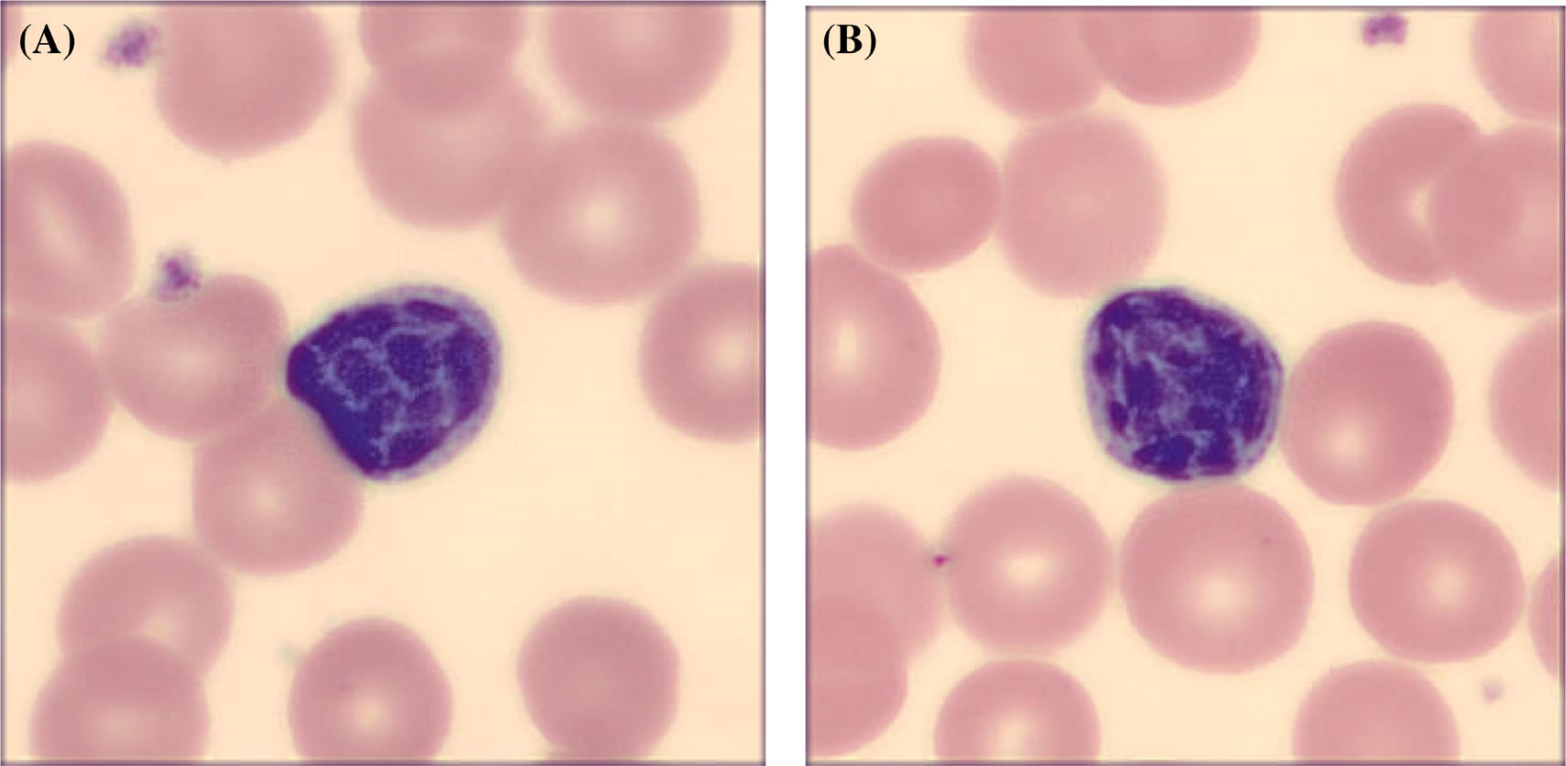
(A,B) Morphology of abnormal lymphocytes demonstrates large clumps of chromatin giving an appearance of “shattered” nuclei and “broken” nuclear margin (1000× magnification)

The treatment was changed to Imbruvica (10/2020) that demonstrated an exceptional response despite utilizing substandard dose of 140 mg per day. A 4‐month follow‐up PET scan (01/2021) showed metabolic resolution of the nodes above and below the level of diaphragm, and bony lesions with Lugano score of 2 (2PS). Interestingly, 1 week before commencing Imbruvica, the patient developed absolute lymphocytosis 7.48 × 10^9^/L that, 3 months later, peaked at 98.0 × 10^9^/L consistent with tumor flare. The majority of lymphocytes displayed salient nuclear clumping giving the impression of “shattered” chromatin and “broken” nuclear margin (shown in Figure 3A,B). During the following 10 months, the lymphocytosis has gradually declined reaching the nadir of 2.18 × 10 ^9^/L. Of note, despite the resolution of absolute lymphocytosis, a proportion of the lymphocytes continued to demonstrate atypical morphology. Flow cytometry confirmed an abnormal B‐cell population positive for CD19, CD23 (partial), CD200, CD25 (dim), CD38 (dim) and CD45 (dim) while negative for CD10, CD20, FMC7, CD11c, CD103 and CD123. CD5 expression was partial, dim and ZAP70 expression was negative. Additional studies showed the IGVH gene status to be unmutated and FISH studies were negative for trisomy 12 and deletion of 11q22.3 (ATM), 13q14.3 or 17p13.1 (TP53). The patient remains in remission by clinical and PET criteria 13 months after initiation of Imbruvica and 23 months after the diagnosis.

## 2 DISCUSSION

The aCLL appears to be a relatively rare form of CLL progression. While clinically indistinguishable from non‐accelerated CLL, the expanded proliferation centers in aCLL have been associated with adverse prognostic features, such as elevated LDH, unmutated IGHV configuration, ZAP70 positivity, high‐risk cytogenetics and propensity to more aggressive behavior with shorter survival.^2^ At the same time, neither ZAP70 nor IGHV mutational status could discriminate between aCLL and typical CLL although all tested patients with aCLL (*n =* 9) demonstrated only unmutated IGHV.

The patient under discussion demonstrated several distinctive features that could contribute to our expanding knowledge of aCLL. First, the patient exhibited extensive bone involvement that, in contrast to classical CLL, has not been reported in aCLL. Second, the patient's lymphocytes exhibited the chromatin pattern with a prominent “shattered” appearance reminiscent of Pelger‐Huet‐like dysplastic anomaly.^3^, ^4^ Unfortunately, the cell morphology escaped attention at the time of the diagnosis because of the lack of absolute lymphocytosis, but such peculiar morphology is believed to be an inherent characteristic of malignant cells rather than the effect of Imbruvica. Third, the lack of overt peripheral lymphocytosis at the outset was consistent with the diagnosis of a lymphoma. Whether this was an additional distinctive feature of this case is not clear because the original study did not specify if the 12% of the CLL population without overt peripheral blood involvement developed into actual aCLL.^2^ Fourth, the lack of CD20 expression was atypical and unexpected because lymphocytes in proliferation centers typically demonstrate higher CD20 expression.^2^ Fifth, although prognosis of aCLL compares unfavorably with the common CLL demonstrating median survival from the time of biopsy of only 34 months,^2^ the outlook in this patient remains unknown. It is noteworthy that, following a suboptimal response to CHOP, she demonstrated an excellent response to Imbruvica, which was remarkable because Bruton's tyrosine kinase inhibitors have yet to demonstrate their utility in aCLL. Aside from the two recently reported cases,^5^ the lack of experience with Imbruvica in aCLL prompted the administration of the reduced dose (140 mg/day), but even such a substandard dose triggered a pronounced peripheral lymphocytosis (tumor flare) that, similarly to “non‐accelerated” CLL, was transient.

The increased variant allele fraction of the *TP53* mutation (84%) (Table 1) may be secondary to second allele loss due to mutation, chromosomal deletion involving the 17p13 locus, or loss of heterozygosity.^6^ Clinically, mutations in *TP53* and *SF3B1* have been associated with inferior progression‐free survival in the setting of typical CLL.^7^ At the same time, the former has shown a predictive value of ibrutinib compared to chemoimmunotherapy in CLL patients.^8^

In conclusion, this case of aCLL characterized by several unusual features that could potentially distinguish it as a novel, rare variant of aCLL. Because peripheral lymphocytosis was lacking at diagnosis, the case was compatible with SLL subsequently evolving into leukemic phase. The patient also exhibited extensive and unique bone involvement but, despite the advanced disease, showed an indolent asymptomatic course. In addition to CD20‐negativity, the striking chromatin clumping in malignant lymphocytes has not been reported previously. Finally, in contrast to the standard chemotherapy, this aCLL case demonstrated exceptional sensitivity to Imbruvica by responding to substandard dose. Further studies with attention to clinical and morphological details could determine whether this case represents a peculiar, hitherto undescribed form of aCLL.

## CONFLICT OF INTEREST

The author declares no conflicts of interest.

## AUTHOR CONTRIBUTION

Leonid L. Yavorkovsky identified the case, acquired, analyzed and interpreted the clinical data including the morphology of the tumor cells, reviewed literature, and wrote and approved the manuscript.

## ETHICS STATEMENT

Ethics approval was not required for this study. A written informed consent was obtained from the patient for publication of the details of their medical case and any accompanying images.

## Data Availability

The data that support the findings of this study are openly available in the Division of Oncology at Kaiser Permanante San Jose Medical Center. All the data generated or analyzed during this study are included in this article. All the datasets on which the conclusions of the paper rely available to editors, reviewers and readers. Further enquiries can be directed to the corresponding author.
